# Subtypes and Prognosis of Guillain-Barré Syndrome in Southwest China

**DOI:** 10.1371/journal.pone.0133520

**Published:** 2015-07-22

**Authors:** Gang Zhang, Qi Li, Rongrong Zhang, Xiao Wei, Junyi Wang, Xinyue Qin

**Affiliations:** 1 Department of Neurology, The First Affiliated Hospital of Chongqing Medical University, Chongqing, China; 2 Department of Medical Technology, Chongqing Medical and Pharmaceutical College, Chongqing, China; Hannover Medical School, GERMANY

## Abstract

The proportion of different subtypes of Guillain-Barré syndrome (GBS) and their prognosis varied significantly among different regions. This study attempts to investigate the clinical subtypes and outcome of GBS in southwest China. Patients with GBS admitted to The First Affiliated Hospital of Chongqing Medical University from January 2006 to March 2013 were included in our study. Patients were classified into acute inflammatory demyelinating polyneuropathy (AIDP) group, acute motor axonal neuropathy (AMAN) group, Miller-Fisher syndrome (MFS) group, cranial nerve variants(CNV), Bickerstaff's brainstem encephalitis overlaps with GBS (BBE-GBS) group and unclassifiable group based on clinical features and electrophysiological findings. Hughes function grade score (HFGS) was used to assess the prognosis at 3 and 6 months. The prognosis of different subtypes and outcome predictors were analyzed. The most common subtype of GBS was AIDP (57%), followed by AMAN (22%) and MFS (7%). The prognosis of AMAN and BBE-GBS is similar at 3 month(P = 0.0704)and 6 month (P = 0.1614) follow-up. The prognosis of AMAN group was poorer than that of AIDP group at 3 month and 6 month follow-up (P<0.001). Outcome of MFS group and that of CNV group at 6 months were both good(Hughes≤1). Hughes≥3(P<0.0001,OR = 6.650,95%CI = 2.865 to 15.023))and dysautonomia (P = 0.043,OR = 2.820,95%CI = 1.031 to 7.715)) were associated with poor outcome at 6 month follow-up. AIDP is the most common subtype of GBS. Prognosis of AMAN group and BBE-GBS group is poorer than that of AIDP group at 3 month and 6 month follow-up. Hughes≥3 at nadir and dysautonomia are predictors of poor prognosis at 6 month follow-up.

## Introduction

Guillain–Barré syndrome (GBS) is a common neurological disorder that is characterized by symmetrical weakness of the limbs, which reaches a maximum severity within 4 weeks [1,2]. Although intravenous immunoglobulin (IVIG) and plasma exchange (PE) were proven to be effective treatment options for GBS [3–5], many patients still have poor prognosis and sequelae such as decreased mobility, severe long-term fatigue syndrome and chronic pain.[6] The reported mortality of GBS varies between 3% and 7%[7–9]. Typical GBS[10] is an acute, predominantly motor neuropathy involving distal limb paresthesias, relatively symmetric leg weakness, and frequent hyporeflexia or areflexia. Recent study suggests that some patients with GBS had normal or hyperreflexia[11,12].According to a recent classification, Bickerstaff's brainstem encephalitis (BBE) can also be included into GBS variants [13]. Previous reports from western countries showed that acute inflammatory demyelinating polyneuropathy (AIDP) is the most common subtype of GBS [14,15], while reports from northern China showed that acute motor axonal neuropathy (AMAN) is the most common subtype of GBS in China [16].The proportion of different subtypes of GBS and their prognosis varied significantly among different regions [17–21].

To date, the subtypes and outcome predictors of GBS remains unknown in southwest China. We set up this study to investigate the clinical characteristics and outcome predictors of GBS in southwest China.

## Materials and Methods

### Patients

Institutional review board approval and informed consent were obtained. Patients with GBS who were admitted to the First Affiliated Hospital of Chongqing Medical University between January 2006 and March 2013 were included into our study. The patients were classified into the following five categories as follows: acute inflammatory demyelinating polyneuropathy (AIDP) group, acute motor axonal neuropathy (AMAN) group, Miller-Fisher syndrome (MFS) group, cranial nerve variant (CNV), Bickerstaff's brainstem encephalitis overlaps with or without Guillain-Barre syndrome (BBE-GBS) and the unclassified group based on clinical features and electrophysiological findings. The unclassified group included pure sensory neuropathy of 3 cases, sensory and motor axon neuropathy of 3 cases, relapses of 2 cases.

### Clinical and Electrophysiological examinations

Hughes functional grading scale (HFGS) was used for evaluating the severity of the disability[22] as follows: 6-death; 5-need mechanical ventilation; 4-bedbound; 3-walk with aid; 2-walk without aid; 1- run with minor deficit; 0- normal. Patients with a HFGS equal to or more than 3 points were defined as severe GBS. Patients with a HFGS less than 3 points were defined as mild GBS. Good prognosis was defined as HFGS≤1 at 6 month follow-up. Poor prognosis was defined as HFGS>1.

Nerve conduction velocity (NCV) was performed using standard procedures. A value was defined as abnormal if it was outside the normal laboratory range, corrected for age. Patients were classified as AMAN or AIDP on the basis of the electrodiagnostic criteria reported by Hadden and his colleagues [17].

### Statistical Analysis

Statistical analysis was performed by using SAS software (version 9.2). Classification variables analysis was performed with the use of Chi-square test or the exact probability method. Test level of α was corrected by Bonferroni correction in comparisons among multiple groups. Repeated Measures was used to compare the mean HFGS at nadir,3 months and 6 months follow up among different subtypes. Univariate analysis was used to identify the factors associated with poor prognosis, which were further analyzed by Logistic regression analysis for predictors independently related to the poor prognosis. A p value less than 0.05 was considered statistically significant.

## Results

### Baseline Clinical Characteristics

A total of 170 patients who diagnosed with GBS were included into our study. The clinical characteristics of each GBS subtype were listed in Table 1. There were 110 (65%) males and 60 (35%) females. The mean age of the patients was 47 years (age range14–82 years). History of antecedent infection or vaccination was observed in 49% of patients 1 to 4 weeks before the onset of GBS, which include 67 cases of upper respiratory infection,11 case of diarrhea,1 case of zoster herpes,1 case of pneumonia, 1 case of injection of tetanus vaccine, 1 case of urinary tract infection. Severe GBS was observed in 142 patients (84%). The remaining 28 patients (16%) had mild GBS. A total of 30 patients had mechanical ventilation due to respiratory failure. Thirteen (7.6%) patients died during hospitalization. Patients with GBS received either intravenous immunoglobulin or plasma exchange as needed.

**Table 1:** Clinical characteristics of each GBS subtype (n,%).

Subtype [n (%)]	AIDP	AMAN	MFS	CNV	BBE-GBS		
	n = 97	n = 37	n = 12	n = 8	n = 8	χ2-value	P-value
Serious	91(94)	36(97)	0	1(13)	7(87)	101.867	0.000
MV	11(11)	14(38)	0	1(13)	4(50)	18.12	0.000
Precursor infection	40(41)	22(59)	6(50)	3(37)	5(63)	4.457	0.384
Male	61(63)	24(65)	10(83)	7(88)	7(88)	4.513	0.341
Age≥40y	61	27	7	7	7	4.580	0.333
Bulbar paralysis	27(28)	14(38)	3	6(75)	6(75)	9.713	0.046
Bilateral facial palsy	18(19)	8(22)	4	5(63)	7(88)	24.809	0.000
Urinary retention	14(14)	8(22)	0	1(12)	1(12)	1.193	0.755
Arrhythmia	7(7)	10(27)	0	2(25)	3(38)	12.834	0.005
Sense disorder	46(47)	12(32)	6(50)	3(38)	2(27)	3.854	0.426
Pain	32(33)	7(19)	3(25)	1(12)	2(27)	3.768	0.438
PE or IVIG	64(66)	25(68)	10(83)	3(38)	4(50)	5.389	0.250

Comparisons of clinical characteristics among subtype groups by chi-square, α was corrected using Bonferroni correction in chi-square of multiple comparisons, *α*’ = 0.005.

### Comparisons of the HFGS among subgroups at nadir

The HFGS was significantly higher in patients with AMAN than those with AIDP (P = 0.027)); The HFGS was not significantly different between AIDP group and BBE-GBS group(P = 0.236); The HFGS was not significantly different between AMAN group and BBE-GBS group(P = 0.966). Incidence of patients needing mechanic ventilation between BBE-GBS group and AMAN group had no significant difference(P = 0.523), Incidence of mechanic ventilation was significantly higher in patients with BBE-GBS group than those with AIDP group (P = 0.003).Incidence of mechanic ventilation was significantly higher in AMAN group than AIDP group (P = 0.000). The HFGS score in different subgroups at different stages were illustrated and compared in Table 2 and Table 3.

**Table 2:** Mean value of HFGS score in different subgroups at different stages (mean± SD).

HFGS score time	Mean value of each subtype				
	AIDP(*n* = 73)	AMAN(*n* = 35)	AMFS(*n* = 12)	CNV(*n* = 7)	BBE-GBS(*n* = 7)
nadir	3.71±0.88	4.40±1.06	1.67±0.71	2.00±0.58	4.57±1.40
3months	1.20±1.18	1.54±1.45	1.67±0.71	0.57±0.53	2.67±1.75
6months	0.62±0.88	1.21±1.17	0.72±0.79	0.29±0.49	2.00±1.67

**Table 3:** Comparisons of HFGS score in different subgroups at different stages.

	nadir		3 months		6 months	
	F-value	P-value	F-value	P-value	F-value	P-value
AIDP/AMAN	4.961	0.027	10.332	0.001	15.264	0.000
AIDP/MFS	14.083	0.000	0.746	0.398	0.257	0.612
AIDP/CNV	8.565	0.004	0.871	0.352	0.952	0.330
AIDP/BBE-GBS	1.408	0.236	13.582	0.000	13.210	0.000
AMAN/MFS	23.147	0.000	6.994	0.009	7.415	0.007
AMAN/CNV	14.862	0.000	5.744	0.017	7.623	0.006
AMAN/BBE-GBS	0.000	0.966	3.291	0.070	1.973	0.161
MFS/CNV	0.000	0.966	0.054	0.831	0.217	0.638
MFS/BBE-GBS	12.967	0.000	12.228	0.001	10.351	0.001
CNV/BBE-GBS	9.745	0.002	10.733	0.001	10.732	0.001

### Prognosis & Predictors

The results of univariate analysis of outcome predictors of patients with GBS were listed in Table 4. Patients with AIDP has significantly better outcome at 3 months follow up and 6 months follow up than patients with AMAN (P = 0.001; P = 0.000,respectively) and patients with BBE-GBS (P = 0.000; P = 0.0001,respectively); Prognosis of AMAN group and BBE-GBS group at 3 months follow up (P = 0.070)and 6 months follow up(P = 0.161) had no significant difference. Prognosis of MFS group and that of CNV group at 6 months were both good(HFGS≤1).The results of predictors of prognosis were showed in Table 5.

**Table 4:** Univariate analysis of outcome predictors of patients with GBS (n, %).

Impact factors	Good prognosis	Poor prognosis	χ2-value	P-value
	(n = 92)	(n = 47)		
Male	58(63)	34(72.3)	0.201	0.273
Age>60years	42(45.7)	29(61.7)	3.207	0.073
Summer & fall	42(45.7)	22(46.8)	0.017	0.897
Precursor infection	43(46.7)	25(53.2)	0.518	0.472
Diarrhea	5(5.4)	5(10.6)	1.262	0.261
Bulbar paralysis	24(26.1)	26(55.3)	11.754	0.001
Bilateral facial palsy	17(18.5)	17(36.2)	5.270	0.022
Dysautonomia	22(23.9)	28(59.6)	17.177	0.000
Peak time≤14days	78(84.8)	39(83.0)	0.076	0.083
Nadir HFGS≥3	70(76.1)	46(97.9)	10.692	0.001
Pain	20(21.7)	9(19.1)	0.126	0.722
Numbness	45(48.9)	20(42.6)	0.505	0.477
IVIG/PE	60(65.2)	33(70.2)	0.351	0.554
Axonal injury	16(18.0)	20(44.4)	10.262	0.001
Diarrhea	5(5.4)	5(10.6)	1.262	0.261

**Table 5:** Logistic regression analysis of the predictors at 6 month after GBS.

Related factor						OR 95% CI	
	B	S.E	Wals	P	OR	Lower limit	Upper limit
Dysautonomia	1.0367	0.5135	4.0755	0.0435	2.820	1.031	7.715
Nadir HFGS≥3	1.8810	0.4227	19.7999	< .0001	6.560	2.865	15.023
Constant	-8.4265	1.6893	24.8830	< .0001			

## Discussion

GBS includes many subtypes such as AIDP, AMAN, FS, BBE-GBS, etc. AIDP and AMAN was referred frequently in the previous reports [15,17,23]. Report from northern China suggest that AMAN accounts for approximately 65% of patients with GBS [16]. In another study, Ye Y, et al reported that AMAN accounts for 33% in northeast China [23].Strikingly, data from European and north America suggested that AMAN constitute only 1 to 3 percent of GBS [15,17]. In our study, we reported that 22% of GBS cases are AMAN in southwest China. Consistent with reports from other parts of China, we found that the AMAN subtype is more common in China. However, AMAN only accounts for 22% of cases in southwest China, suggesting that significant geographical distribution of AMAN in China. Emerging evidences from immunological and clinical studies [24–26] suggested that patients may develop AMAN after campylobacter jejuni enteritis. Therefore we postulated that the geographical variations of campylobacter jejuni infection among different regions and countries may contribute to the strikingly different distribution of AMAN.

In our study, we found that MFS or MFS-GBS account for 7% of GBS cases. In sharp contrast, Mitsui Y et al reported 26% of GBS patients are MFS-GBS in Japan [14]. Similarly, Ng YS and colleagues demonstrated that MFS constitute 25% of GBS in Singapore [19]. We also found that both CNV and BBE-GBS is up to 5% in our study. A retrospective study of 43 patients reported that both MFS and BBE constitute 7% of GBS patients, CNV count for 5% of GBS patients in Taiwan[27].

In our study, mild patients only account for 16% of cases. We found that 84% of patients with GBS are severe cases. These findings are consistent with reports from northeast of China, which demonstrated that up to 80% of cases were severe GBS [23].The severe GBS constitute 67% in Europe [20] and 42% in United States [21]. Our findings demonstrated that the proportion of severe GBS is higher in China than that of Europe and United States.

The severity of patients in different GBS subtype groups are quite different. The ratio of critically-ill patients in the AMAN group is higher than that of AIDP group in northeast China[23]. Sundar and colleagues reported that patients with axon injury were prone to have respiratory failure [28]. The data present here shows that the mean HFGS of AMAN group and ratio of mechanic ventilation were significantly higher than that of AIDP group. Patients in the CNV group were almost always mild cases. Only one patient among the eight patients of the CNV group need mechanic ventilation. The patient recovered completely. The result of our research shows that patients with AMAN group and BBE-GBS are usually severe cases and are more likely to require mechanic ventilation than other subtypes of GBS.

According to a report of 132 case study from northeastern China, the prognosis of AIDP group is similar to that of AMAN group at 6 moth follow-up [23].Another study from England suggested that axonal lesion pattern is associated with poor prognosis [28]. Of 139 patients were followed up in our research, prognosis of AMAN group was poorer than that of AIDP group at 3 month and 6 month follow-up.

A recent study from Japan showed that patients with MFS usually had good recovery and had no residual symptoms [29]. In another study of 65 patients from south China, the authors demonstrated that patients with MFS may have good recovery and these patients were not treated with immunomodulating therapy [30]. In the present study, twelve MFS patients were all completely recovered at 6 month follow-up. Our findings corroborates with previous reports from Japan and southern China.

Patients with cranial nerve variant of GBS usually have good prognosis. An Indian study involving 38 patients with cranial never palsies showed that cranial nerve palsies recovered completely at the end of 3 month after onset of symptoms[31]. Our study shows that patients with pure cranial nerve palsies had good prognosis at 6 month after onset of symptoms. All 7 patients with cranial nerve variant have complete recovery at 6 month follow-up.

GBS was once considered as a disease that only affected periphery nerve. The recent discovery of anti-GQ1b antibodies in patients with GBS, MFS and BBE provided important evidence that these disorders formed part of the same disease spectrum[32]. A recent study revealed that anti-GQ1b antibodies were present in 83% of patients with MFS and 68% of patients with BBE[27], suggesting that central nervous system can also be involved in GBS. In a study of 62 patients in Japan[33], 75% of the 37 patients with BBE-GBS have good prognosis at 6 moth follow-up and 25% have residual weakness. However, only 2 of 7 patients with BBE-GBS in our study have a good prognosis. The discrepancies between the prognosis of patients with BBE-GBS in the present study and that of earlier studies suggest that substantial geographical variation of GBS prognosis. Our findings suggest that the prognosis of BBE-GBS and AMAN are worse than that of patients with AIDP.

We also found that HFGS at nadir equal or exceeded 3, dysautonomia are independent predictors of poor outcome at 6 month follow-up. A study from India also show that peak disability, dysautonomia are related to the poor recovery at 3 months [34].A high grade on the GBS Disability Scale at neurological examination, diarrhea preceding GBS onset [35–38] and advanced age are all predictors of poor outcome[6,7,35,37].

In summary, this report is the first time provide the data on GBS subtypes and outcome in southwest China. Further studies are needed to clarify the underlying mechanism of regional variation in GBS subtypes and outcome in China.
